# Endogenous Repair and Regeneration of Injured Articular Cartilage: A Challenging but Promising Therapeutic Strategy

**DOI:** 10.14336/AD.2020.0902

**Published:** 2021-06-01

**Authors:** Hongzhi Hu, Weijian Liu, Caixia Sun, Qiuyuan Wang, Wenbo Yang, ZhiCai Zhang, Zhidao Xia, Zengwu Shao, Baichuan Wang

**Affiliations:** ^1^Department of Orthopaedics, Union Hospital, Tongji Medical College, Huazhong University of Science and Technology, Wuhan 430022, China.; ^2^Department of Gynecology, General Hospital of the Yangtze River Shipping, Wuhan 430022, China.; ^3^Department of Nephrology, Xiangyang Central Hospital, Affiliated Hospital of Hubei University of Arts and Science, Xiangyang 441100, China.; ^4^Centre for Nanohealth, ILS2, Swansea university Medical school, Swansea, SA2 8PP, UK.

**Keywords:** articular cartilage injury, endogenous cartilage regeneration, matrix microenvironment, mesenchymal stem/progenitor cells, chondrocytes

## Abstract

Articular cartilage (AC) has a very limited intrinsic repair capacity after injury or disease. Although exogenous cell-based regenerative approaches have obtained acceptable outcomes, they are usually associated with complicated procedures, donor-site morbidities and cell differentiation during *ex vivo* expansion. In recent years, endogenous regenerative strategy by recruiting resident mesenchymal stem/progenitor cells (MSPCs) into the injured sites, as a promising alternative, has gained considerable attention. It takes full advantage of body’s own regenerative potential to repair and regenerate injured tissue while avoiding exogenous regenerative approach-associated limitations. Like most tissues, there are also multiple stem-cell niches in AC and its surrounding tissues. These MSPCs have the potential to migrate into injured sites to produce replacement cells under appropriate stimuli. Traditional microfracture procedure employs the concept of MSPCs recruitment usually fails to regenerate normal hyaline cartilage. The reasons for this failure might be attributed to an inadequate number of recruiting cells and adverse local tissue microenvironment after cartilage injury. A strategy that effectively improves local matrix microenvironment and recruits resident MSPCs may enhance the success of endogenous AC regeneration (EACR). In this review, we focused on the reasons why AC cannot regenerate itself in spite of potential self-repair capacity and summarized the latest developments of the three key components in the field of EACR. In addition, we discussed the challenges facing in the present EACR strategy. This review will provide an increasing understanding of EACR and attract more researchers to participate in this promising research arena.

Articular cartilage (AC) injury is a common disease that usually caused by sport injuries, accidental trauma or joint diseases [1]. Once injured, AC has a very limited self-repair ability [2]. Even small injuries would progress to larger lesions over time if left untreated, and eventually lead to osteoarthritis (OA) [3]. AC injuries are often result in severe knee pain, swelling and joint stiffness, which seriously affect patient’s quality of life. The medical costs associated with the treatment of AC injuries have been increasing due to the high prevalence around the world [4]. Biological repair of injured AC may significantly reduce these costs by restoring the healthy native tissue and providing long-term symptom control.

Exogenous cell-based approaches, including autologous chondrocyte implantation (ACI) [5], and application of various mesenchymal stem/progenitor cells (MSPCs) either alone [6] or in combination with scaffolds [7, 8], have been developed for injured AC repair, and acceptable therapeutic outcomes have been obtained. However, these methods are usually associated with complicated procedures, donor-site morbidities and less controllable regulation during ex vivo cell expansion [9, 10]. Endogenous regenerative approaches by recruiting resident MSPCs into the injured sites take full advantage of the body's own regenerative potential to achieve tissue repair and regeneration while avoiding the aforementioned drawbacks [11]. Through initiating endogenous regenerative mechanisms, a range of tissues, such as adipose, bone, tendon, etc., have been successfully regenerated [12-14].

Microfracture is the most commonly applied surgical technique that triggers the migration of endogenous mesenchymal stem cells (MSCs) from bone marrow to injured regions to regenerate AC tissue [15]. However, the neo-tissues are mostly comparatively weak fibrous cartilage relative to native hyaline cartilage [16]. The reasons for this failure could be attributed to an inadequate number of recruiting cells and adverse local tissue microenvironment after AC injury [17]. A strategy that improves local matrix microenvironment and recruits a large number of endogenous cells into the injured sites might enhance the success of endogenous AC regeneration (EACR) [18, 19]. In this review, we discussed: 1) what is the endogenous self-repair potential of AC and what are the regenerative limitations in AC self-repair? 2) what are the latest developments of the three key elements (endogenous stem cells, chemoattractants and scaffolds) in the field of EACR? 3) what are the challenges facing in the present EACR strategy? The objective of this review is not only to give readers an increasing understanding of the present EACR strategy, but also to attract more researchers to participate in this promising research arena with the aim of exploiting more effective AC regenerative approach.

## Endogenous self-repair otential of AC

In almost all tissues, there is a resident population of mesenchymal stem/progenitor cells (MSPCs) [20]. These cells exist inside stem-cell niches which maintain the state of quiescence, self-renewal or active differentiation of MSPCs [21]. They could undergo directional migration under appropriate stimuli to maintain tissue homeostasis and repair injured tissues [21, 22]. A resident population of progenitor cells, also referred to as cartilage-derived progenitor cells (CPCs), has been found in the normal and degenerative AC [23]. In addition, some tissue-specific MSPCs also have been found in other areas of the joint including synovium [24], synovial fluid (SF) [25], meniscus [26], infrapatellar fat pad [27], suprapatellar fat pad [28], and perichondrial groove [29], perichondrium [30]. Some previous studies demonstrated that many injured-associated products (such as cell lysates, ECM fragments, high-mobility group box 1, HMGB1 and stromal cell derived factor-1, SDF-1) could stimulate *in vitro* migration of MSPCs [31, 32]. More importantly, an increased percentage of MSPCs-marker positive cells was observed in the injured cartilage tissue in comparison to the normal cartilage tissue [33, 34]. In addition, MSPCs were present in higher numbers in the SF after cartilage injury [35]. All these findings indicate that when AC becomes injured, MSPCs in multiple stem-cell niches surrounding the injured sites would be activated in response to the stimulation of injured signals and migrate into the injured sites to produce replacement cells. Moreover, many *in vitro* and *ex vivo* studies have shown that chondrocytes are also able to migrate under different external stimuli, although *in vivo* chondrocyte migration remains to be further determined [36, 37]. To sum up, an endogenous self-repair attempt exists after AC injury. However, full recovery of the structure and function of the injured cartilage in human adults is rare or even considered to be absent. If cartilage tissue cannot regenerate itself, what are the limitations in injured cartilage self-repair?

## Limitations of endogenous AC self-repair

Endogenous tissue self-repair is a very complicated process, which involves cell migration and extensive crosstalk between the migrated cells and the local tissue microenvironment. The questions arise as to whether endogenous cells can migrate smoothly into the injured sites, whether the number of the migrated cells is sufficient, and what will happen to the migrated cells in the local tissue microenvironment?

### Effect of AC structure and injured stimuli on migration of endogenous cells

**Figure 1.**
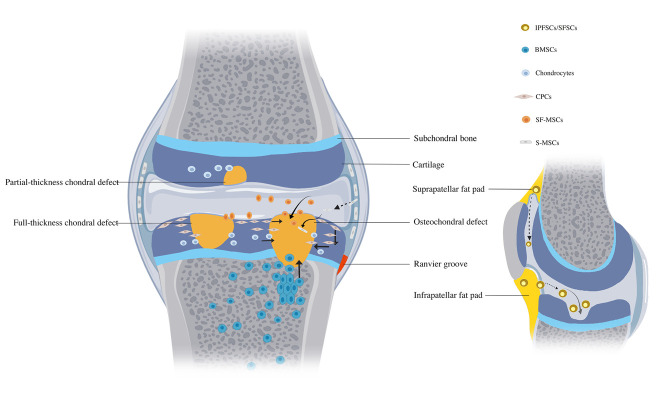
Cell types involved in EACR and their potential migration routes. CPCs, cartilage-derived progenitor cells; IPFSCs/SPFSCs, infrapatellar/suprapatellar fat pad-derived stem cells; BMSCs, bone marrow-derived mesenchymal stem cells; S-MSCs, synovium-derived mesenchymal stem cells; SF-MSCs, synovium fluid-derived mesenchymal stem cells; MPCs, meniscus-derived progenitor cells; RMSCs, Ranvier groove derived mesenchymal stem cells. Depending on the type of AC lesions, MSPCs involved in the repair process might differ. Partial- and full-thickness chondral defects: chondrocytes, CPCs, IPFSSCs/SPFSCs, S-MSCs, SF-MSCs, MPCs and RMSCs (not exhibited in the picture); Osteochondral defect: chondrocytes, CPCs, IPFSSCs/SPFSCs, S-MSCs, SF-MSCs, MPCs, RMSCs and BMSCs.

AC is an avascular tissue that consisted of a dense, well-organized collagen fibrillar network with a low cell-to-matrix ratio [38]. Such a unique structure might hinder cartilage self-healing to a certain degree. Firstly, unlike the tissues with powerful stem-cell niches (such as bone), the cartilage tissue contains a very small number of resident CPCs [38, 39]. The self-repair capacity of AC might be greatly restricted because of the limited number of CPCs available for migration. Secondly, the ECM of AC is relatively dense. The structural feature is essential for the mechanical stability and the proper function of the cartilage tissue [38]. However, it might partly hinder the migration of chondrocytes and CPCs embedded in the ECM. In addition, when the lesion is completely located within the cartilage layer without penetrating the tidemark, the matrix molecules within the remaining hyaline cartilage, such as dermatan sulfate and other proteoglycan, can inhibit cell migration and adhesion [40]. Lastly, AC does not contain blood vessels that are critical for tissue repair [38]. For partial- and full-thickness chondral defects (Fig. 1), the nutrients and regulatory molecules required for tissue repair and regeneration are only obtained by diffusion through normal cartilage and SF, and are therefore very limited [38, 41]. Also, due to the absence of blood vessels, there might be no immediate-early repair response with monocytes and macrophages to injured cartilage [42]. Therefore, the avascular nature of AC may also explain in part lack of cartilage regeneration.

The weak natural recruitment signals might also be partly responsible for the failure of endogenous AC self-repair. As mentioned above, the injured cartilage tissue can release a large number of injured-associated products. They, as recruitment signals, can stimulate surrounding chondrocytes and multiple MSPCs to migrate into the injured sites to produce replacement cells [31, 32]. However, these recruitment signals are normally too limited to recruit sufficient endogenous cells to result in successful regeneration of injured AC [43].

### Potential effects of local tissue microenvironment on migrated cells

AC injuries, either acute injury (such as sport injury and trauma) or chronic injury (such as OA), usually cause substantial changes in local tissue microenvironment [44, 45]. These changes can significantly influence cell survival, proliferation and differentiation. In such cases, even if the number of the migrated cells is sufficient, it is difficult to repair the injured AC. A good understanding of the local tissue microenvironment is of great significance for us to exploit more effective tissue regenerative approaches.

The (sterile) inflammation response plays a critical role in tissue healing [46]. When AC is injured, the injured tissue will release damage-associated molecular patterns (DAMPs), such as HMGB1 and S1008/9 [47-49]. These DAMPs subsequently induce the surrounding cells (such as chondrocytes, MSPCs and synoviocytes) to release pro-inflammatory chemokines which attract inflammatory cells into the injured sites to trigger the inflammation response [48]. Of note, compared with chondrocytes, CPCs express higher levels of pro-inflammation genes, such as interleukin-6 (IL-6) and IL-8 [50, 51]. Acute inflammatory response after AC injury primarily involves IL-1, IL-6, IL-18 and tumor necrosis factor-α (TNF-α) [45, 52, 53]. The production of these cytokines is not exclusive to cartilage tissue; on the contrary, much of it comes from synoviocytes, adipocytes derived from intraarticular fat pad and circulating immune cells derived from synovial and intramedullary vessels [44]. These inflammatory cytokines significantly inhibit the proliferation and differentiation of MSPCs and chondrocytes [54-57]. Han *et al*. [54] reported that both IL-1 and TNF-α inhibited the expression of chondrogenic-related genes in synovium-derived mesenchymal stem cells (SMSCs). Similar findings were observed in another study by Wehling *et al*. [55], in which both IL-1 and TNF-α inhibited chondrogenesis of human BMSCs in a dose-dependent manner. In addition, Martensson *et al*. [57] found that both IL-1β and TNF-α inhibited differentiation of growth plate chondrocytes.

The chronic cartilage injury, usually caused by OA, is characterized by low-grade inflammation, ECM breakdown and osteogenic microenvironment. Compared with acute inflammation, the chronic inflammatory response involves more inflammatory cytokines. For example, IL-17 is exclusively produced by a group of T helper cell and therefore is primarily involved in OA-associated chronic cartilage injury [52]. In addition to affecting the biological behaviors of cells, these inflammatory mediators also lead to chronic breakdown of the ECM by stimulating the overproduction of aggrecanases, collagenases, tissue plasminogen activator, nitric oxide (NO) and reactive oxygen species (ROS) [58-61]. NO, which is induced by IL-1 and TNF [58], inhibits chondrocyte proliferation and ECM synthesis [59]. Overproduction of ROS results in chondrocyte senescence, death and ECM degradation [60]. Additionally, along with the development of OA, the subchondral bone begins to become more permeable, and some osteogenic cytokines, such as bone morphogenetic proteins (BMPs) and transforming growth factor-β (TGF-β), potentially leak into cartilage tissue [44, 61]. These osteogenic microenvironment favors chondrocyte hypertrophy and osteogenesis [44]. Hypertrophic chondrocytes express type X collagen and some additional molecules, such as matrix metalloproteinase-13 (MMP-13) and vascular endothelial growth factor (VEGF) [62], which substantially alter the pericellular microenvironment of local cell populations.

Tissue engineering approaches that overcome these obstacles might improve and enhance EACR. Currently, the trend is to deliver bioactive factors or anti-inflammatory drugs to regulate local highly inflammatory or osteogenic micro-environment [63]. For example, Wang *et al*. [64] combined collagen scaffold with resveratrol to form an anti-inflammatory scaffold, once implanted in a rabbit osteochondral region, revealed remarkable anti-inflammatory and regenerative properties. However, injured AC is present in a more complicated local tissue microenvironment, more efforts are needed to further understand it.

**Figure 2.**
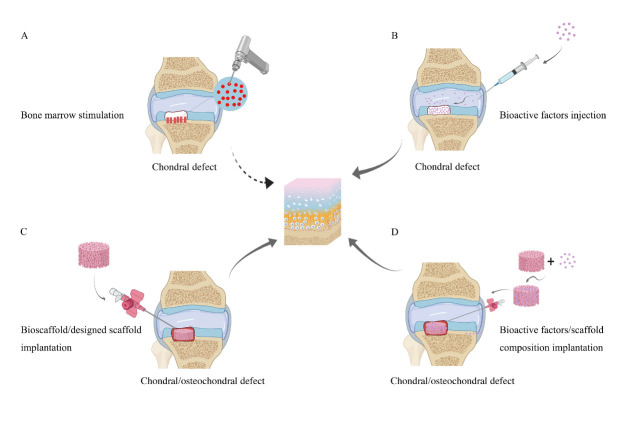
Therapeutic options of the present endogenous chondral/osteochondral regeneration. (A) Bone marrow stimulation; (B) Bioactive factors injection; (C) Bioscaffold/designed scaffold implantation with or without microfracture; (D) Bioactive factors/scaffold composition implantation with or without microfracture.

## AC regeneration based on endogenous regenerative mechanisms

Recently, there is growing evidence demonstrated that endogenous regeneration approach is a very promising, cost-effective alternative for cartilage repair and regeneration [65, 66]. Compared with tissue regeneration based on exogenous cells, it offers greater advantages in terms of handling, cost, time, and regulation. An enhanced endogenous tissue regeneration achieved by tissue engineering technology has largely repaired those injured ACs [67, 68] (Fig. 2) We here systematically reviewed the latest developments of the three key components in the field of EACR?

### Cells for endogenous cartilage regeneration

Endogenous MSPCs play an important role in EACR. On the one hand, they can migrate into the local defect under appropriate stimuli and participate in cartilage repair and regeneration directly. On the other hand, they can also secret bioactive factors (such as growth factors, exosomes, etc.) to influence cartilage regeneration indirectly [63].

### Cell types

Multiple resident MSPCs and abundant chondrocytes are present in or around the injured sites. They can be activated by the injured signals and then migrate into the injured sites to participate in the repair events [69]. These MSPCs mainly include CPCs, BMSCs, SMSCs, SF-derived MSCs (SFMSCs), infrapatellar fat pad-derived stem cells (IPFSCs), suprapatellar fat pad-derived stem cells (SPFSCs), meniscus progenitor cells (MPCs), and MSCs in perichondrium and Ranvier groove [17, 18]. Depending on the type of AC lesions, chondral or osteochondral defects, MSPCs involved in the repair process differs (Fig.1).

## Potential migration routes of endogenous repair cells

### CPCs and chondrocytes

Due to their beneficial localization and innate chondrogenic phenotype, CPCs are considered to be a promising cell source for AC regeneration [70]. Although distributing through the whole cartilage layer, CPCs are mainly located in the superficial zone and specifically express proteoglycan 4 (Prg4) [71]. A lineage analysis in mice demonstrated that these Prg4 expressing-cells would migrate into the deeper layers during the development of cartilage and serve as the progenitor population of all mature chondrocytes [72]. In addition to the vertical migration, CPCs can also migrate horizontally to replenish the stem cell pool and effect a lateral expansion of the AC layer [73]. Therefore, when the AC is injured, CPCs would migrate into the injured sites from vertical and horizontal directions to produce the replacement cells.

Chondrocytes are the most abundant cells within AC. In the past, it is believed that chondrocytes in adult cartilage are unable to migrate due to the surrounding highly tensile ECM [17, 74]. However, a recent study showed that a significant percentage of articular chondrocytes also express alpha-smooth muscle actin, indicating their potential migration ability [75]. More importantly, a growing body of *in vitro* and *ex vivo* evidence supports the migratory potential of chondrocytes [35, 37]. Therefore, these chondrocytes, as a new promising target cell, can be utilized to improve the endogenous regeneration of injured AC. Serial cartilage studies have showed that segmental neo-cartilage was formed by adjacent tissue protruding during AC regeneration [76, 77]. These findings suggest that the chondrocytes around the injured sites would migrate horizontally under the simulation of injured signals and participate in AC defect healing.

### BMSCs

BMSCs, usually as an exogenous seed cell type, were used for cartilage repair and regeneration [78]. In fact, they also have been widely investigated as an endogenous seed cell type in the past three decades [79, 80]. Self-repair of the partial- and full-thickness cartilage defects is rare or even considered to be absent, which might be greatly attributed to the dense subchondral bone plate (SBP) between the cartilage and bone marrow cavity [81, 82]. Although SBP is a thin tissue, it can effectively block BMSCs from migrating into cartilage tissue. The commonly used microfracture technique employs the concept of endogenous BMSCs migration to regenerate the injured cartilage tissue [79]. In this procedure, some holes are created on the injured sites of AC through SBP to the bone marrow cavity, and subsequently BMSCs migrate into the injured sites via these holes under the stimulation of chemotactic signals from the microfracture site. Although the neo-tissues are not as satisfactory as expected, the successful use of this procedure provides sufficient evidence for the potential migration route of BMSCs in EACR.

### Other intraarticular resident MSPCs

As mentioned above, other intraarticular resident MSPCs, such as SMSCs, SFMSCs SPFSCs, IPFSCs, MPCs and MSCs in Ranvier groove, might also involve in the endogenous cartilage regeneration [28, 29, 83-87]. Due to special intraarticular anatomic sites, they exhibit higher chondrogenic potential than those MSPCs from adipose tissues, periosteum and bone marrow. Unfortunately, to date, there is no direct evidences on the migration routes of these intraarticular resident MSPCs. Considering the distance between these stem-cell niches and the injured sites, a possible route is that tissue-resident MSPCs firstly enter into SF, and subsequently migrate into the injured sites (Fig. 1). Some findings also implicitly indicate this potential migration route. For example, Jones *et al*. found that the number of the progenitor cells in the SF significantly increase during acute/chronic AC injury [88]. Of note, the migration route may vary because of the difference between joint morphology in big and small animals [89, 90]. For instance, in rabbit knee joint, synovium tissue extends to the surface of meniscus, which facilitates SMSCs to migrate directly from synovium to the injured sites [89].

## Chemoattractants for endogenous cartilage regeneration

MSPCs recruitment is the first and most important step for endogenous tissue regeneration [11]. MSPCs express a number of receptors for chemokines and growth factors. The ligand-receptor binding activates intracellular signaling pathways (such as JAK/STAT, MAPK, PI-3K/Akt, ERK1/2 and Wnt) to induce or modulate migration of MSPCs [91-93]. The pattern of MSPCs recruitment is chemotaxis, which allows their directional migration along a chemoattractant gradient [94]. In view of the fact, because the natural endogenous chemotactic signals are normally too weak to execute the successful repair and regeneration of many tissues including AC. Approaches by adding additional chemoattractants (such as chemokines and growth factors) to enhance migration of endogenous MSPCs may accelerate and improve endogenous tissue regeneration. Although several previous articles have systematically reviewed these chemoattractants, they set their sights on the whole endogenous regenerative medicine [11, 18]. Of note, chemotactic responses vary among MSPCs isolated from different tissue types [94, 95]. Hence, we here summarized those chemoattractants which were specifically used for EACR (Table 1). In addition, the potential side effects of these chemoattractants are also shown in this table.

**Table 1:** Chemoattractants for endogenous cartilage regeneration.

Chemoattractants (Ligands)	Chemoattractants (Receptors)	Evidence of migration of chondrocytes or MSCs induced by various chemoattractants	Potential side effects
Chemokines			
SDF-1(CXCL12;)	CXCR4	Homing BMSCs and facilitating their chondrogenic differentiation *in vitro and in vivo* [76, 96].	Inhibiting the migration of human subchondral mesenchymal progenitor cells *in vitro* [97]. Inducing subchondral bone deterioration by erroneous recruitment of MSCs [98].
IL-8 (CXCL8;)	CXCR1,2	Recruiting autologous BMSCs to the injured site of articular cartilage [99].	Inducing articular chondrocyte hypertrophy [100, 101].
MCP-1 (CCL2;)	CCR2	Inducing directional migration of various adult stem/progenitor cells [102, 103].	Inhibiting the chondrogenic differentiation of MSCs in vitro [104].
MIP- 3α (CCL20;)	CCR6	Triggering the homing of BMSCs for cartilage repair *in vitro and in vivo* [99].	Inducing osteoclast formation and osteoblast proliferation [105].
SCM-1 (lymphotactin/XCL1)	XCR1	Recruiting the stem cell migration from the subchondral bone [97].	-
Growth factors			
TGF-β1	TGF-βR	Promoting endogenous MSCs recruitment [106].	Inducing synovial proliferation, fibrosis inflammatory responses and osteophyte formation [107-109].
TGF-β3	TGF-βR	Enhancing endogenous stem cell recruitment and facilitating in situ articular cartilage regeneration [110].	-
BMP-2	BMPRIs, BMPRIIs	Recruiting endogenous MSCs to regenerate injured cartilage [111, 112].	Causing osteogenic differentiation and osteoblast growth [44]. Inhibiting the cartilage repair response [113].
BMP-4	BMPRIs, BMPRIIs	Recruiting endogenous MSCs to regenerate injured cartilage [111].	-
BMP-7	BMPRIs, BMPRIIs	Recruiting endogenous MSCs to regenerate injured cartilage [111].	Inhibiting MSCs proliferation [114].
PDGF	PDGFRa/b (CD140a/b)	Promoting recruitment of endogenous progenitor cells and chondrocytes *in vivo* [111, 115, 116].	Involved in atherosclerosis, fibrotic conditions, as well as malignancies [117].
IGF-1	IGF-1R	Promoting MSCs and chondrocytes homing and recruitment [118-120].	Inducing hypoglycemia, seizures, jaw pain, myalgia, edema, headaches, increased liver and kidney mass, altered liver function, erythema and lipohypertrophy at the injection-site [121-123].
FGF-2	FGFR-1 (CD331), -2 (CD332), -3 (CD333), -4 (CD334)	Contributing to the migration of the BMSCs and chondrocytes [113, 124].	Inducing inflammation and osteophyte formation when used alone [125].
NGF	NGFR	Showing the promigration effect for CSPCs [126].	Stimulating both the growth of tumor cells and angiogenesis [127].
HGF	HGFR (c-Met)	Exerting an important role in chondrocyte migration and cartilage remodeling [128, 129].	Involved in osteophyte formation under certain circumstances [130].
MGF	-	Facilitating the recruitment of endogenous stem cell for cartilage regeneration [110].	-
Other factors			
PRP	-	Enhancing the migration and stimulated the chondrogenic differentiation of MSCs [131-133].	Causing allergy reaction [134].
BMC	-	Facilitating recruitment of MSCs and chondrocytes [135].	-
MSCs-derived exosomes	-	Enhancing the migration of chondrocytes [136, 137].	-
LPP	BMP-RII	Stimulating the site-directional migration of CPCs *in vitro* [138].	-
Platelet lysate	-	Supporting the migration of both chondrocytes and MSCs [139].	-
FN	Integrin ±5β1	Enhancing the proliferation, migration, and chondrogenic differentiation capacity of CPCs [140].	-

* Although many other factors (such as interferon inducible protein, IP-10; thymus and activation-regulated chemokine, TARC; B-lymphocyte chemoattractant, BLC; etc.) also have the ability to facilitate MSCs migration and tissue repair, they are not discussed in this review. In our study, we only focus on those chemoattractants that have been shown to contribute to EACR. *MSCs* Mesenchymal stem cells; *SDF-1* Stromal cell derived factor; *BMSCs* Bone marrow mesenchymal stem cells; *IL* Interleukin; *MCP* Monocyte chemoattractant protein; *MIP* Macrophage inflammatory protein; *SCM* Single C motif; *TGF-β* transforming growth factor beta; *BMP* Bone morphogenetic protein; *PDGF* Platelet-derived growth factor; *IGF* Insulin-like growth factor; *FGF* Fibroblast growth factor; *NGF* Nerve growth factor; *CSPCs* Cartilage stem/progenitor cells; *HGF* Hepatocyte growth factor; *MGF* Mechano growth factor; *PRP* Platelet-rich plasma; *BMC* bone marrow concentrate; *SMSC* Synovium-derived marrow mesenchymal stem cells; *LPP* Link protein N-terminal peptide*; CPCs* Cartilage-derived progenitor cells; *FN* Fibronectin.

## Scaffolds for endogenous cartilage regeneration

Along with cell recruitment, another important issue is how to create an appropriate microenvironment for cell residence, differentiation and new tissue formation. Scaffolds play a crucial role in these events. They allow the activated resident MSPCs to migrate into and serve as a temporary “home” for these migrated cells. Meanwhile, they provide specific microenvironment to direct cell differentiation according to the tissues that require repairing [141]. Apart from the aforementioned characteristics, the “perfect” scaffold for EACR should also allow for irregular fill and a good incorporation with surrounding cartilage, and be sufficiently strong to bear normal mechanical stress within the joint during the process of regeneration [142, 143]. In addition, the scaffolds can be implanted in a one-step procedure. In the past decades, a substantial body of studies have been published, in which various scaffolds, either alone or in combination with chemoattractants, have been used for endogenous chondral and osteochondral regeneration *in vitro* and in some *in vivo* models [132, 144, 145]. We here review the different scaffolds that are available for EACR (Table.2).

Although many scaffolds represent themselves as potential candidates in AC regeneration based on exogenous cells, they seem to be powerless in EACR because of the lack of the ability to induce cell homing [170]. By combining these scaffolds with bioactive factors, which promotes endogenous cells to migrate into the scaffolds as well as regulates cell proliferation and chondrogenic differentiation, it is helpful to improve and enhance EACR [143, 159, 171]. Zhang *et al*. [148] created an *in-situ* matrix environment conductive to CPCs and SMSCs migration and adhesion by mixing chemokine SDF-1 and collagen type I, which significantly promoted partial-thickness cartilage defect self-repair in rabbit knee joint. A scaffold system containing chemokines and growth factors might further improve the quality of neo-cartilage by simultaneously promoting cell homing and chondrogenic differentiation. More recently, Chen *et al*. [155] fabricated a novel dual bioactive factor-releasing scaffold, SDF-1α/TGF-β1-loaded silk fibroin-porous gelatin scaffold (GSTS), to enhance the healing of cartilage defect. They found that GSTS facilitated *in vitro* MSCs homing, migration, chondrogenic differentiation, and SDF-1α and TGF-β1 had a synergistic effect on the promotion of *in vivo* cartilage forming. In addition, given that there were substantial differences in regeneration between cartilage and bone, several bilayer or multilayer scaffolds were developed, and their combination with bioactive factors have been used for endogenous osteochondral defect repair [157-159]. Collectively, many bioactive factors have been loaded into different scaffolds to repair and regenerate chondral or osteochondral defects and are summarized in Table 2. In addition, when bioactive factors are loaded into a scaffold, a release rate allowing a sustained therapeutic dose should also be considered [151, 154].

**Table 2:** Scaffolds for endogenous cartilage regeneration.

	Scaffold type	Layers	Animal model	Bioactive factors	Refs
Scaffold + bioactive factors	Poly-epsilon-caprolactone and hydroxyapatite	-	rabbit	TGF-β3	[146]
	CS glycerol-phosphate/blood	-	rabbit	Thrombin (Factor IIa)	[147]
	Type 1 COL scaffold	-	rabbit	SDF-1	[148]
	DBM-chitosan hydrogel	-	rabbit	BMSC specific affinity peptide E7	[149]
	HA-PCL	-	porcine	TGF-β3	[150]
	SF	-	rabbit	TGF-β, MGF	[110]
	Photocrosslinkable hydrogel glue		rabbit	PRP	[151]
	Photoinduced hydrogel glue	-	rabbit	Stem cell-derived exosomes	[152]
	3D printed silk-fibroin-gelatin Scaffold	-	rabbit	BMSC affinity peptide	[144]
	PLGA		rabbit	PRP	[132]
	Acellular cartilage matrix	-	rabbit	SAP-bone marrow homing peptide	[66]
	Fibrin/hyaluronan hydrogel	-	mouse	AntimiR-221	[145]
	SF/HA-tyramine hydrogel	-	rabbit	Aptamer (Apt19s)	[153]
	PEO-PPO-PEO thermosensitive hydrogel	-	minipig	rAAV-sox9	[154]
	Extracellular matrix	-	rabbit	Stem cell-derived exosomes	[136]
	GSTS	-	rat	SDF-1α/TGF-β	[155]
	COL	Bilayer	rabbit	PRP	[131]
	COL	Bilayer	rabbit	BMP-4	[156]
	COL-silk scaffold	Bilayer	rabbit	PTHrP	[157]
	OSA/NSC-PCL/PEG-fibre-SA/nano HA	Multilayer	rabbit	FGF-2, BMP-2, TGF-β1, LIPUS	[158]
	PLGA/polylysine heparin-COL/CS/HAS	Bilayer	rabbit	Kartogenin, TGF-β1	[159]
Bioscaffold/ designed scaffold	Non-woven multifilamentous	-	ewes	N/A	[160]
	CS-glycerol phosphate	-	rabbit	N/A	[161]
	PLCL	-	rabbit	N/A	[143]
	PGA	-	sheep	N/A	[162]
	Porous PLGA	-	rabbit	N/A	[163]
	PLA-PCL	-	rabbit	N/A	[164]
	Methacrylated HA-PLGA	-	rabbit	N/A	[165]
	Decellularized cartilaginous ECM	-	rabbit	N/A	[166]
	Oriented pores cylindrical PLGA	-	rabbit	N/A	[167]
	3D printed PLCL-aggrecan	-	rabbit	N/A	[142]
	Acellular cartilage sheets	-	swine	N/A	[168]
	Acellular bone matrix	-	minipig	N/A	[68]
	HA-based hydrogels	-	mouse	N/A	[116]
	COL/microporous electrospun nanofiber	Bilayer	rabbit	N/A	[169]

*PLCL* Polylactic acid poly-ε-caprolactone; *PGA* Polyglycolic acid; *PLGA* Poly (lactide-co-glycolide); *PLA* Polylactic acid; *PCL* Poly (∈-caprolactone); *ECM* Extracellular matrix; *HCF* Heparin-conjugated fibrin; *HA* Hyaluronan; *PEO* Poly (ethylene oxide); *PPO* Poly (propylene oxide); *GSTS* SDF-1α/TGF-β loaded SF-porous gelatin scaffold; *OSA* Oxidized sodium alginate; *NSC* N-succinyl chitosan; *PEG* Polyethylene glycol; *SA* Sodium alginate; *COL* Collagen; *CS* Chitosan; *SF* Silk fibroin; *HAS* Hyaluronic acid sodium; *TGF* Transforming growth factor; *MGF* Mechano growth factor; *SAP* Self-assembling peptide; *SDF* Stromal cell-derived factor; *PRP* Platelet-rich plasma; *PTHrP* Parathyroid hormone-related protein; *BMP* Bone morphogenetic protein; *DBM* Demineralized bone matrix; *FGF* Fibroblast growth factor; *rAAV* recombinant Adeno-associated virus.

Some bioscaffolds alone, either native matrices or biomimetic materials, have the potential to recruit endogenous cells and do not require additional supplement of bioactive factors to exert beneficial effects [164, 165]. One good example of such bioscaffolds is the acellular/decellularized ECM (a/dECM) [68, 166]. They can not only mimic the natural tissue matrix environment in which cells reside and function, but also have the capacity to promote cell homing because of the various intrinsic growth factors contained in this environment. Xue *et al*. [168] found that acellular cartilage sheets alone could induce endogenous host cells migration and achieve generally satisfactory repair of cartilage defects. Instead of using whole dECM, some individual ECM proteins might also exert good functions. Vainieri *et al*. [116] reported that hyaluronic acid-based hydrogel alone supported endogenous cell infiltration and provided an amenable microenvironment for cartilage production. In addition, some specifically designed scaffolds exhibit potent potentials in EACR. Dai *et al*. [167] reported that the oriented macroporous PLGA scaffold promoted the migration of endogenous cells and successfully induced endogenous osteochondral defect regeneration. Other studies with similar design also obtained satisfactory outcomes [160, 161]. The use of three-dimensional (3D) bio-printing technology allows for more complex designs, which can precisely control the internal microstructure (such as pores and microchannel) of the scaffold, and therefore might provide a more suitable microenvironment for EACR [136, 144]. Recently, Guo *et al*. [142] used the 3D bio-printing technology to fabricate a functionalized scaffold (PLC-aggrecan), and they found that the 3D-printed scaffold had great potential to improve the quality of cartilage regeneration.

## Challenges facing in the present EACR strategy

The regenerative approaches by enhancing the recruitment of endogenous cells have successfully regenerated the injured cartilage in many *in vivo* animal models [154, 162]. Although these results are exciting, only a scarce amount of methods have been able to move from the bench to the bedside [172, 173]. There are still many challenges and concerns that need to be addressed before their clinical application.

Numerous studies demonstrated that both chondrocytes and various MSPCs derived from multiple stem-cell niches surrounding the injured sites had great potential to be ideal candidates for EACR [115, 174]. However, almost all studies focus on one or even two cell types, which is a far cry from reality. As shown in Figure 1, EACR is a complicated process involving various cell types. How these migrated cells interact with each other and which type of cells plays the decisive role in EACR remain unclear [95]. For engineering endogenous cell recruitment, one of the most challenges is the selection of effective chemoattractant(s). Although many chemoattractants have potent chemotactic activities for MSPCs *in vitro* [175, 176], it is difficult to identify which one is the most appropriate chemoattractant. Firstly, the chemotactic responses vary among MSPCs isolated from different tissue types [94, 95]. Secondly, since most bioactive factors have multiple effects, exposure of MSPCs to a chemoattractant may stimulate many collateral responses (Table.2) in addition to the chemotaxis desired. Moreover, in a majority of the studies, MSPCs are typically exposed to one or two bioactive factors [110, 124], which is hard to simulate the complicated internal multiple signals. In the field of biomaterials, some scaffolds alone significantly support cell recruitment *in vitro* and regenerate cartilage tissue *in vivo* with some success [68, 116]. However, how the components and architecture of these scaffolds affect cell recruitment and cartilage regeneration are still unclear. The exploration of these potential mechanisms will be helpful for the design of the next-generation engineering scaffolds. Furthermore, the emerging 3D bio-printing technology allows for fabricating personalized scaffolds with controlled internal micro-architecture structures [136, 142]. Theoretically, 3D-printed scaffolds have great potential for the application in EACR. However, more researches are needed to find the best suitable bio-inks.

In addition, (sterile) inflammation is inevitable after cartilage injury. Therefore, the effects of inflammation on EACR should be taken into account. However, most of previous studies seem to have ignored and weakened the roles of inflammation and inflammatory factors during cartilage regeneration [42, 177]. Also, the local inflammatory microenvironment in the common cartilage defect models are not entirely consistent with those in patients with cartilage injuries, especially for OA patients [147, 150, 178]. Some improved *in vitro* and *in vivo* model systems that more closely resemble the actual inflammatory microenvironment in the damaged joint should be developed.

## Conclusion

Despite certain challenges still exist, EACR is a promising, cost-effective strategy for injured cartilage. It can successfully repair the injured cartilage while avoiding exogenous regenerative approach-associated limitations. More importantly, it circumvents the complex processes involved in exogenous tissue regeneration, and thereby facilitates the clinical translational. The increasing understanding of the poor self-repair mechanisms underlying AC, the latest developments of EACR and the challenges facing the present EACR will help researchers to explore problem-solving effective regenerative approaches. An interdisciplinary strategy that bridges tissue engineering with cell biology, biochemistry, physiology, and material science might further optimize EACR.
